# Quantitative imaging for early detection and risk stratification of cardiovascular disease using 4D flow MRI and arterial spin labelling

**DOI:** 10.6026/9732063002001769

**Published:** 2024-12-31

**Authors:** Bassam Alkhalifah

**Affiliations:** 1 Department of Radiology and Medical Imaging, College of Medicine, Qassim University, Buraydah, Saudi Arabia

**Keywords:** Cardiovascular disease, 4D flow MRI, arterial spin labelling, hemodynamic, blood flow dynamics, tissue perfusion, atherosclerotic plaques

## Abstract

Heart failure (HF) significantly burdens global healthcare, necessitating early detection and precise risk stratification. Advanced imaging techniques like 4D flow Magnetic resonance imaging (MRI) and arterial spin labelling (ASL) provide crucial insights into cardiac function by capturing complex flow patterns and measuring myocardial blood flow. Hence, this study explores how these modalities can enhance early detection and risk assessment of cardiovascular diseases, aiming to improve patient outcomes. Ten patients aged ≤ 65 with clinically compensated cardiomyopathy were recruited. MRI examinations included 4D flow MRI using a 1.5 T Philips Achieva Scanner and ASL imaging on a 3 Tesla scanner. Data analysis for 4D flow MRI involved segmenting the left ventricle and categorizing pathlines into flow components, while ASL data were analyzed using Buxton's model to quantify myocardial blood flow (MBF). The study population had a mean age of 49 ± 14 years, predominantly female (6:4). Average heart rate was 61 ± 11 bpm and blood pressures averaged 122/77 mmHg. Left ventricular end-diastolic volume was 179 ± 33 mL with an ejection fraction of 42 ± 5%. Patients showed lower direct flow volume and kinetic energy in early diastolic phases compared to healthy individuals. In conclusion, 4D flow MRI and accelerated ASL is effective for early detection and risk stratification in cardiovascular disease, offering enhanced cardiovascular assessment and potential improvements in patient care.

## Background:

Heart failure (HF) is the final stage of the cardiovascular disease spectrum and a major contributor to the costs associated with healthcare worldwide. There is an urgent need for early detection and accurate risk stratification of heart failure (HF), as the prognosis for people with this condition is sometimes worse than that of several prevalent malignancies [1]. One of the main characteristics of HF is cardiac remodelling, which changes from adaptive to maladaptive stages and raises the risk of symptoms and death [2]. Even patients with early or mild HF, who may appear clinically compensated with normal left ventricular stroke volume (LVSV), exhibit impaired prognosis and clinical outcomes compared to healthy individuals [3]. Heart and artery blood flow is essential to cardiovascular health and changes in intra-cardiac flow patterns have been linked to cardiomyopathy [4]. Advanced imaging techniques such as 3-D, time-resolved flow encoded MRI, or 4D flow MRI, provide comprehensive velocity data, capturing the complex flow patterns within the heart [5]. Recent advancements have enabled the measurement and separation of different flow components in the left ventricle (LV) over time, providing important insights into how the heart functions normally and in disease [6]. In addition, arterial spin labelling "ASL" is a modern MRI technique that measures blood flow in tissues without using contrast agents. While traditionally used for cerebral blood flow quantification, ASL shows great promise for cardiac applications [7]. This technique has been employed to measure myocardial blood flow (MBF) in both animal models and humans, demonstrating compatibility with pharmacological stress testing and the potential for detecting ischemic heart disease [8]. Hence, the potential of 4D flow MRI and ASL in the early detection of cardiovascular disease and the assessment of associated risks was investigated. By examining their principles, applications and clinical impacts, it is intended to highlight how these advanced imaging modalities can enhance cardiovascular assessment and improve patient outcomes.

## Materials and Methods:

## Study population:

Ten patients treated for cardiomyopathy were included in this study. On clinical examination, myocardial pathology was determined by symptoms and signs of heart failure, systolic dysfunction and ECG evidence of ventricular hypertrophy, reduced ejection fraction and mitral annulus depression. Patients were included from cardiology centers. Patients aged 65 years and older with cardiomyopathy and patients who were not candidates for MRI were excluded from the study. The study was approved by the ethics committee in accordance with the Declaration of Helsinki and all the patients gave written informed consent.

## Data acquisition 4D flow:

All subjects underwent MRI to obtain morphological images and 4D blood flow profiles. 4D blood flow data were acquired during free breathing using an electrocardiogram-triggered, retrograde navigation-gated, 3D, time-resolved phase-contrast MRI sequence on a Clinical 1.5 T Philips Achieve scanner. It was done to acquire and post-process of 4D flow data. The following were included in the scan parameters: k-space segmentation factor = 2, echo duration = 3.7 ms, repetition time = 6.3 ms, flip angle = 8°, parallel imaging (SENSE) speed-up factor = 2 and velocity encoding (VENC) = 100 cm/s. With these parameters, 50.4 ms of temporal resolution was achieved. The field-of-view (FOV), which covered the left heart in each patient, was adjusted and the spatial resolution was 3 x 3 x 3 mm^3^. The average scan time for the Cartesian 3D cine phase contrast sequence, including navigator efficiency and arrhythmia rejection was 31 minutes (range: 16-57, median: 30, standard deviation: 8).With navigator efficiency and arrhythmia rejection excluded, the mean time needed acquire all k-space lines for the 4D flow data was about 12 minutes (std: 2, median: and12 and range: 9-18). Following post-processing and the application of the Eriksson *et al.* described data quality control procedures, simultaneous gradient field, phase-wrap and background phase error adjustments were made. Path lines that depicted blood flow were continually drawn during this procedure, beginning at a certain point in the left atrium continuing through the heart cycle. The route of an imagined massless particle through the time-resolved velocity field is represented by a pathline. Path lines that exited the blood pool at points where real blood flow cannot occur, including through the ventricular apex, were indicative of errors in the data. This quality control was passed by all data sets. Using the video steady-state free motion sequence, morphological long-axis and two groups of short-axis images were acquired over 30 times while the subject held his/her last breath. The slice thickness was 8 mm. The acquired and reconstructed pixel sizes for short-axis images were 2.19 x 1.78 mm2 and 1.37 x 1.37 mm2, respectively.

## Imaging methods for ASL:

A Signa Excite HDxt 3 Tesla scanner with an 8-channel cardiac receiver coil was used for all imaging procedures. Myocardial ASL was done using a short-axis mid-ventricular slice using flow-sensitive alternating inversion reversal "FAIR". The system has two different pulse types: one for image control and one for image marking. The fat saturation module, snapshot-balance steady-state free precession (b-SSFP) image acquisition and a 19-TR Kaiser-Bessel ramp for flip angles to reduce transients. The 19-TR Kaiser-Bessel ramp reduces the flip angle. The use of ASL scanning was performed as described by Zun *et al.* [9], imaging parameters included 3.2 ms TR, 1.5 ms TE, 50° flip angle, 62.5 kHz receiver bandwidth and 24-26 cm isotropic field of view (FOV). Slice thickness was 10 mm and a 30 mm thick inversion plate was used to control the image to verify image location. The scan matrix size used was 96 x 96, resulting in a 307 ms acquisition window before and after fuel saturation and ramp modules, giving a total imaging window of 440 ms. In accelerated ASL, 2 sensitivity coding reduction (SENSE) is used, resulting in a 153 ms acquisition window and a 286 ms total display window. Use breath hold and heart rate to reduce spurious energy. The FAIR marker reverse pulse was timed to mid-diastole using plethysmograph gating and determined to be 77% of the RR interval. The seven breath holds in the ASL procedure took two to three minutes to complete. For the purpose of estimating blood equilibrium magnetisation (M0) and thermal noise (TN), the first breath-hold recorded baseline and noise pictures. Following that, six consecutive 12-second breath-holds were used to obtain six pairs of control and tagged photos, with a 6-second gap between each image. This procedure held true for both expedited and reference scans. A localisation scan and the identification of a mid-short axis slice marked the start of the scan process. Correct slice location free from banding artefacts was guaranteed by a baseline image that did not contain the FAIR labeling pulse. A frequency scout scan was carried out to determine the ideal offset frequency if artefacts were detected. In order to estimate the coil sensitivity map, a fully sampled baseline image was then obtained during a 1-second breath-hold. In every subject, the accelerated ASL scan came after the reference ASL scan.

## Data analysis:

## 4D flow MRI:

The left ventricle "LV" was manually segmented from end-diastolic "ED" and end-systolic "ES" short-axis images using the utility Segment. The segmentation of the ED was resampled to obtain a volume with isotropic voxels matching the flow data. A line is created from the center of each voxel in the LV segmentation and traced forward and backward in time until the ES appears earlier or later, respectively. This line represents the total LV end-diastolic volume "EDV" recorded during a cycle. Depending on the location of each pathway relative to the ventricle determined by the ES segmentation, four different flows were defined in the ES: direct flow, retained input, delayed ejection flow and residual segment. In addition, direct entry and intermittent entry are also divided into early entry and late entry [10]. The times from the beginning of diastole to mid-diastole and from mid-diastole to ED are determined as early and late diastole, respectively. The frame with the smallest line along the mitral valve plane is accepted as the mid-diastole time. Each path liner presents a volume of blood with a calculable mass using the density of blood (ρ blood=1060kg/m^3^) and a known velocity at every time point. The kinetic energy (KE) for each path line was calculate date very time point using the formula, KE=1/2ρ [11] V {pathline} v {pathline}2, where-V{pathline} is the volume represented by one pathline and v{pathline} is the velocity of the pathline at a given time. The KE for each flow component was the sum of the KE of its pathlines and this KE was normalized by the component's volume. The KE of each component's pathline's at ED was compared.

## ASL data analysis:

The study used accepted techniques described by Zun *et al.* [9] to calculate noise levels and myocardial blood flow (MBF). MATLAB was used for all data processing (Math Works, Natick, MA). After image reconstruction, the myocardium was manually segmented using the spatiotemporal averaging filter and resampled to polar coordinates. Buxton's general kinematic model served as the basis for MBF quantification.

## MBF = [C - T/2M0 .TI .exp(-TI/T1)]:

The control photos have an average cardiac signal of (C), while the tagged images have an average cardiac signal of (T). The post-labeling delay time (TI) equals the R-R interval and the baseline image signal is (M0). Blood's longitudinal relaxation time (T1) is assumed to be 1650 ms. there were two options: the number of resampled segments and the size of the spatial filter. Using a single segment and a filter size of 2 π, global MBF quantification was done. The filter size for regional MBF quantification was (π/3) with six segments, while the filter size for septal MBF quantification was (2π/3) with three segments. These measurements were contrasted with previous physiological noise assessments.

## Results:

## 4D flow MRI:

## Patient's characteristics:

The demographic and clinical data from 10 dilated cardiomyopathy patients. Females outnumber males 6:4 in these patients with a mean ± SD age of 49 ±14 years. Patients with myocardiopathy have a mean ± SD heart rate of 61±11 bpm. There was average 122 mmHg for systolic and 77 mmHg for diastolic BP. These patients had a left ventricular (LV) end-diastolic volume of 179 mL on average, with an SD of 33 mL and detailed characteristics shown in Table 1.

## LV stroke volumes:

The LV stroke volumes (LVSVs) in the cardiomyopathy group (inflow volume: 77 ± 18 mL; ejected volume: 77 ± 19 mL, respectively). However, the direct flow volume was lesser in cardiomyopathy people (35 ± 11 mL), which meant that the ratio of direct flow to total inflow was also smaller in people with cardiomyopathy (46 ± 9%). The kinetic energy (KE) of blood flow was calculated over time for all components (Figure 1 - see PDF).

To assess the impact of atrial contraction, the early and late stages of DC flow were separated. The ratio of end-diastolic DC flow to total flow reduced in the cardiomyopathy group (P < 0.001), although the ratio of early diastolic DC to total flow remained constant across all groups. In healthy persons, DC flow KE/mL was lower at early diastolic sub-volume in ED (P = 0.003), however there was no difference in diastolic time in patients with cardiomyopathy (Figure 2A - see PDF).

The characteristics of direct flow during the early (E) and late (A) periods of diastole, (Figure 2B - see PDF) compare early and late inflow. The direct flow volume at the end of diastole (A) is given as a percentage of the total intake for both E and A. The direct flow to total inflow ratio for the duration of the diastolic filling phase (cardiomyopathy: 46+9%) can be obtained by adding the values at E and A. (B) The energy in motion at locations A and E. The mean and standard deviation are the data's visual representations.

## LV residual volume:

Although the DCM group had a notably higher EDV (P = 0.021), there was no difference in the proportion of residual volume to EDV between DCM patients and healthy individuals. Compared to healthy persons, DCM patients' residual volume at ED had a considerably greater KE/mL (Figure 1 - see PDF).

## Arterial spin labelling (ASL):

Global myocardial blood flow measured from the reference method was 1.20 ± 0.44 ml/g/min and from the accelerated method was 1.24 ± 0.25 ml/g/min, with no significant difference between the two techniques (P = 0.7297). The physiological noise (PN) was significantly lower in the accelerated method (0.08 ± 0.05 ml/g/min) compared to the reference method (0.20 ± 0.08 ml/g/min, P = 0.0059). Thermal noise (TN) was slightly higher in the accelerated method but within expected ranges due to the shortened readout time and g-factor losses. Measurement of global MBF from all subjects indicated in Figure 3 (see PDF).

## Discussion:

Individuals with compensated HF were treated using a 4D flow MRI technique. The study's 4D flow MRI technique allows for the accurate division of the entire left ventricular (LV) volume into discrete functional components. A thorough comparison of volume, timing and kinetic energy (KE) comparing individuals with compensated heart failure is made easier by this methodology. Despite little LV remodeling brought on by cardiomyopathy. The composition of SV varied, nevertheless, with HF patients having less direct flow in a single cardiac cycle. HF patients had a lower direct flow volume, but by the end of diastole, their KE per millilitre was the same as that of the healthy subjects [10]. Recirculating residual volume made up a similar percentage of total end-diastolic volume in both groups; however, in patients with cardiomyopathy, the residual fluid had a greater KE per millilitre. Furthermore, compared to patients with cardiomyopathy, a higher percentage of direct flow was seen in the late diastolic inflow in normal LVs. Furthermore, Rickter *et al.*'s research, which supports our findings, demonstrates that there are notable variations in LV function and remodelling [11, 12]. Hove and colleagues' research suggests that the normal interaction between blood flow and heart development in the womb promotes continuous adjustment, resulting in positive blood flow [13]. The pathophysiology of HF is likely influenced by similar reactions to flow-induced stresses. Incoming blood can have four different effects on the KE: it can move blood already in the LV, transform into potential energy in the myocardium, dissipate as heat, or decelerate, increasing the LV diastolic pressure (suggested by Sigfridsson *et al.*) [14]. Normal diastolic function depends on the LV experiencing less flow deceleration. Studies using computational fluid dynamics indicate that the KE of early diastolic vortices can stop pressure rises that impede input. As velocities decrease, higher KE of intra ventricular blood resulting from changed LV characteristics (*e.g.*, increased stiffness or poorer relaxation) may cause heightened filling pressures. The four functional flow components during diastole varied in sizes and energy between myopathy and normal LVs. The various flow and energy patterns demonstrate how kinetic energy (KE) influences diastole. Patients with cardiomyopathy have a substantially reduced direct flow volume as compared to normal. Therefore, even though the end-diastolic KE per milliliter is the similar in myopathy LVs, the overall direct flow KE at end-diastole is lower. This means that ejection will require greater systolic contraction in patients with cardiomyopathy. For example, in patients with cardiomyopathy, the end-diastolic retained flow is higher than the KE per milliliter, indicating a greater effect on blood flow, volume and myocardial turnover. In individuals with cardiomyopathy, the end-diastolic KE per millilitre of the direct flow is larger for the early diastolic component when the inflow is divided into early and late diastolic phases. While Stewart *et al.* produced results that were similar, Furthermore, cardiomyopathy patients have a lower fraction of direct flow after atrial contraction, which suggests a compromised diastolic-systolic coupling [15]. This disrupted atrial contraction, which is frequently associated with worsening symptoms of heart failure, may impair KE preservation. Target heart rates and pacing tactics for patients with heart failure may be influenced by knowledge of how late diastolic inflow affects left ventricular outflow.

The pathways taken by the delayed ejection flow and retained inflow are altered by the residual volume surrounding the LV chamber's border. The quantity of non-exchanging residual volume is similar across the categories, however individuals with cardiomyopathy had larger exchanging components (delayed ejection flow and held inflow). Carlhall *et al.*'s research, which indicates that decompensated HF patients had higher residual volumes than compensated patients, is consistent with this [16]. According to this study, individuals with cardiomyopathy had greater KE per milliliter of residual volume during end-diastole. Their myocardium is less flexible and more rigid, which explains this. Furthermore, preventing thrombus and lowering the rise in diastolic blood pressure may be achieved by converting the motion of incoming blood to residual volume motion. Low speeds in large residual volumes could facilitate the formation of a ventricular thrombus. According to Maze *et al.*'s echo-Doppler research, individuals with cardiomyopathy who have LV thrombus had lower top-level blood velocity than patients who do not have thrombus [17]. Sensitivity encoding (SENSE) in ASL has the potential to reduce physiological noise (PN) in cardiac ASL and decrease the acquisition window. This result validates our theory that the primary source of PN is motion during image acquisition. After reducing the acquisition window to about 150 ms per cardiac cycle, motion artefacts in the reference method's control images were noticeably reduced. This implies that motion during the acquisition window creates discrepancies in the reconstructed control and tagged picture pairs, leading to variations in the myocardial blood flow (MBF) measurements over time in the reference technique. These discrepancies were less likely to occur when the acquisition window was shortened with parallel imaging, which resulted in a 60% reduction in PN. This decrease in PN is equivalent to a 158%increase in temporal signal-to-noise ratio (tsnr), directly improving sensitivity to MBF. The finding of our study is supported by the Allen *et al.* [18].

Moreover, MBF values obtained with the accelerated approach were similar to those obtained with the reference method. The significant reduction in physiological noise (PN) is a major factor in lowering the total noise level, even though the increase in true noise (TN) with parallel imaging may seem paradoxical. Even though there is a tiny increase in actual noise, this trade-off leads to an improved overall noise reduction. This implies the possibility of raising acceleration to reduce noise even more until PN and TN gradually drop and match. Although this study included only healthy patients at rest, we expect that the fast ASL approach will improve myocardial ASL sensitivity in both resting and critically ill patients. An early study by Zun *et al.* showed significant PN overall in patients [9]. Using SENSE to shorten the exposure window would also be beneficial when taking images at high heart rate and during sleep. SENSE can't fully unwrap all aliasing when the field of view is smaller than the signal-producing region, which results in residual artefacts. This is just one of SENSE's limitations. An improved and more dependable option is generalised auto-calibrating partially parallel acquisition (GRAPPA), which performs better in tiny fields of view (FOV). In comparison to other methods, the GRAPPA's capabilities provide better image quality and dependability, according to the study by Grinwald *et al.* [19]. It is also worthwhile to investigate fast imaging techniques based on constrained reconstruction and/or partial Fourier analysis. Reduced SNR is the main adverse effect of parallel imaging, which is brought about by g-factor losses and shorter readout times. In contrast to first-pass techniques, temporal SNR limits cardiac ASL approaches. This study demonstrates that they could gain from the higher temporal SNR of parallel imaging. To provide reliable observations, the ASL method depends on a number of crucial assumptions. These include perfect inversion efficiency for all inversion pulses, baseline image intensity as a surrogate for M0 with an assumed blood-tissue partition coefficient of one and a steady heart rate during each breath hold. The accurate measurement of myocardial blood flow and the interpretation of ASL data depend on these essential presumptions. In vivo measurements confirm inversion efficiency continuously above 94%, as demonstrated by the work by Kober *et al.* On the other hand, lengthier labelling delays would require inversion thickness changes [20].

## Limitations:

Meticulous quality control was required, with potential faults arising from elements such as temporal/spatial resolution, VENC and MRI hardware. Data from regions containing aliasing artifacts was excluded. Mismatches owing to patient movement were reduced. The findings are based on a limited cohort, who limits the ability to estimate residual volume and may yield different results under other situations.

## Conclusion:

Our study highlights the valuable role of 4D flow MRI and accelerated ASL techniques in detecting cardiovascular disease early and stratifying risks. Accelerated ASL techniques demonstrated comparable global myocardial blood flow measurements to reference methods with significantly reduced physiological noise, enhancing the reliability of MBF quantification. These findings signify the utility of advanced imaging modalities in providing comprehensive cardiovascular assessments.

## Figures and Tables

**Table 1:** Demographic and clinical data

**Parameter**	**(n = 10)**
Gender (female:male)	6:04
Age (years)	49 ±14
Weight (kg)	82 ± 18
Heart rate (bpm)	61 ± 11
Systolic BP (mmHg)	122 ± 14
Diastolic BP (mmHg)	77 ± 9
LV end-diastolic volume (mL)	179 ± 33
LV sphericity index	0.75 ± 0.12
LV ejection fraction	42 ± 5
LV diastolic function according to echo Doppler indices	
Normal diastolic function	5
Relaxation abnormality	2
Pseudo normal filling	1
Restrictive filling	2
Medication	
ARB/ACE-I	10
Beta-blocker	10
Diuretic	3
Aldosterone I	2
Statin	3
Warfarin	4
Aspirin	2
Digoxin	1
